# Epidemiology of pediatric sepsis in the pediatric intensive care unit of king Abdulaziz Medical City, Jeddah, Saudi Arabia

**DOI:** 10.1186/s12887-021-02686-0

**Published:** 2021-05-07

**Authors:** Mohamed O. Humoodi, Mona A. Aldabbagh, Maher M. Salem, Yousef M. Al Talhi, Sara M. Osman, Mohammed Bakhsh, Abdullah M. Alzahrani, Maha Azzam

**Affiliations:** 1 Department of Pediatrics, King Abdulaziz Medical City, P.O. Box 65362, Jeddah, 21556 Saudi Arabia; 2 King Abdullah International Medical Research Centre, Jeddah, Saudi Arabia; 3 King Saud bin Abdulaziz University for Health Sciences, P.O. Box 65362, Jeddah, 21556 Saudi Arabia

**Keywords:** Mortality, Septic shock, Sepsis, intensive care units, Pediatrics, Critical care

## Abstract

**Background:**

Pediatric sepsis remains a significant cause of morbidity and mortality worldwide. This study aimed to identify the incidence of sepsis and septic shock among patients admitted to the pediatric intensive care unit (PICU) of a tertiary center in Saudi Arabia.  Patients' demographics and risk factors associated with sepsis-related mortality were also investigated.

**Methods:**

A retrospective cohort study was conducted in the PICU of King Abdulaziz Medical City, Jeddah (KAMC-J). KAMC-J is a tertiary care hospital in the western region of Saudi Arabia. A total of 2389 patients admitted to the PICU of KAMC-J between January 1, 2013 and December 31, 2017 were screened and evaluated for sepsis using The Third International Consensus Definitions for Sepsis and Septic Shock (Sepsis-3).

**Results:**

Of the 2389 total admissions to the PICU, 113 patients (4.9%) met the definition of Sepsis-3; 50.4% of the 113 patients met the definition of septic shock. Most patients (66.3%) were less than 6 years old, and 52.2% were male. Eight-five patients (75.2%) had underlying comorbidities. The respiratory system was the most common primary site of infection (57.5%). Bacterial and viral infections were the most common infectious etiology with reported rates of 29.2 and 21.2%, respectively. The median duration of PICU stay was 8 days and the 28-day PICU mortality rate was 23.9%. A Pediatric Sequential Organ Failure Assessment (pSOFA) Score greater than four and a pre-existing percutaneous central venous catheter were associated with a significant increase in mortality, with adjusted odds ratios of 3.6 (95% confidence interval: 1.30–9.93) and 9.27 (95% confidence interval: 1.28–67.29), respectively.

**Conclusions:**

The incidence of sepsis in our institution is comparable to that reported internationally; however, the mortality rate is higher than that of developed countries. Nationwide studies identifying sepsis epidemiology are needed to improve the outcome of pediatric sepsis. Following international guidelines for central-line insertion and maintenance is of paramount importance.

**Supplementary Information:**

The online version contains supplementary material available at 10.1186/s12887-021-02686-0.

## Background

Sepsis is a systemic illness caused by an infectious agent invading the body, which induces the release of inflammatory mediators, resulting in systemic inflammatory response syndrome (SIRS). SIRS is not unique to sepsis. It is a non-specific inflammatory process that can occur after trauma, infection, burns, pancreatitis, and many other diseases [1, 2]. The American College of Chest Physicians/Society of Critical Care Medicine Consensus Conference held in 1991 defined sepsis as SIRS associated with a suspected or proven infection [2, 3]. The International Pediatric Sepsis Consensus Conference accepted this definition for pediatric patients after some modifications. Pediatric SIRS was defined as the presence of ≥2 SIRS criteria, one of which must be abnormal temperature or white cell count. The criteria for vital signs were adjusted according to different pediatric age groups. Suspected or proven infection could be of bacterial, viral, fungal, or rickettsial origin [3].

Sepsis remains a significant cause of morbidity and mortality worldwide. Infectious diseases account for approximately 60% of deaths in children younger than 5 years old [4]. The 2015 SPROUT Study, which included 128 pediatric intensive care units (PICUs) across 26 countries, showed a sepsis point prevalence of 8.2%, with an overall mortality of 25%. PICU mortality varied among regions: 21% in North America, 29% in Europe, 32% in Australia/New Zealand, 40% in Asia, 11% in South America, and 40% in Africa [5].

Knowledge regarding the disease is the first step to fully comprehend the impact of sepsis. A limited number of studies have described the epidemiology of pediatric sepsis in Saudi Arabia and the Middle East and North Africa region [6–10]. Understanding the local epidemiology is necessary to develop guidelines that will increase the early identification and improve the management of sepsis. Therefore, this study aimed to identify the incidence of sepsis and septic shock among patients admitted to the PICU of a tertiary center in Saudi Arabia and investigate patients' demographics and risk factors associated with sepsis-related mortality.

## Methods

### Study design and participants

This was a retrospective cohort study conducted at the PICU of King Abdulaziz Medical City-Jeddah (KAMC-J). All pediatric patients admitted to the PICU between January 1, 2013, and December 31, 2017 were identified. The PICU of KAMC-J is a tertiary unit with a 14-bed capacity and an annual admission of approximately 500 patients. Various respiratory and hemodynamic support measures, including extracorporeal membrane oxygenation, are available for use. This study was approved by the Institutional Review Board of King Abdullah International Medical Research Center prior to commencement.

The study included pediatric patients aged younger than 15 years who had been admitted to the unit during the defined period and met the definition of Sepsis-3. Medical records were reviewed, and all patients were screened and evaluated for sepsis. Patients with missing medical records or incomplete data were excluded.

### Study definitions

In this study, sepsis was diagnosed using the Sepsis-3 definition instead of the SIRS criteria. Sepsis-3 defines sepsis as life-threatening organ dysfunction due to a dysregulated host response to infection using the Pediatric Sequential Organ Assessment (pSOFA) Score (Additional file 1) [11]. This score assesses six organ systems: respiratory, hematological, hepatic, cardiovascular, neurological, and renal. A subscore of 0–4 points is calculated for each component. The pSOFA score was calculated for all patients at and 24 h after the time infection was first suspected. Patients with confirmed or suspected infection and an increase of ≥2 points in the pSOFA score were diagnosed with sepsis. The lowest value of each subscore during the abovementioned period was used to calculate the pSOFA score.

Septic shock was clinically defined as sepsis with persistent hypotension (requiring vasopressors) in conjunction with a serum lactate level ≥ 2 mmol/L, despite adequate fluid resuscitation [11].

### Data collection and analysis

Variables of interest included demographic data, underlying medical conditions, presence of central lines (either surgical [tunneled] or percutaneous [peripherally inserted central (PIC)]), site of infection, causative organisms, and patient outcome. The primary outcome was 28-day mortality from the date of sepsis onset. The secondary outcome was 7-day mortality from the date of sepsis onset. Patient data were collected from the computerized database and paper files.

Data were analyzed using descriptive statistics to evaluate the study population.  Categorical variables were described as total numbers and percentages. Quantitative variables were described as median and interquartile range.

Univariate logistic regression was used to determine the factors associated with an increased likelihood of mortality. The dependent variable was 28-day mortality from the date of sepsis onset. Variables that reached statistical significance in the univariate model subsequently underwent multivariate analysis to estimate the adjusted odds ratios (OR) and 95% confidence intervals (CI). Statistical analysis was performed using SPSS version 26.00 (IBM Corp., Armonk, NY, USA). Statistical significance was set at *p* < 0.05.

## Results

During the study period, 2389 patients were admitted to the PICU; 2260 were excluded because they did not meet the sepsis diagnosis criteria and 16 were excluded due to missing data. Ultimately, 113 patients (4.96%) met the definition of sepsis and were enrolled. Most septic patients (66.3%) were less than 6 years old, and 47.8% were female. An acute raise (> 4 points) in pSOFA score since admission was observed in 45.1% of patients. The most common primary site of infection was the respiratory tract (57.5%). Table 1 details the demographic and clinical characteristics of the patients with sepsis.

**Table 1:** Demographics and clinical profile of patients admitted to the Pediatric Intensive Care Unit (PICU) with sepsis

Variables	Septic patients ***n*** = 113
**Median age in months (IQR)**	31 (86)
**Age groups (%)**	
• < 1 year	36 (30.9)
• 1 to < 6 year	40 (35.4)
• 6–14 years	37 (32.7)
**Female gender (%)**	54 (47.8)
**Source of admission (%)**	
• Inpatient wards	62 (54.9)
• Emergency room	47 (41.6)
• Other local hospitals	3 (2.7)
• Outpatient department	1 (0.9)
**Comorbidities (%)**	
• None	28 (24.8)
• Malignancy	33 (29.2)
• Chronic Respiratory Disease	8 (7.1)
• Cardiovascular Disease	5 (4.4)
• Immunodeficiency	3 (2.7)
• Neuromuscular Disease	16 (14.2)
• Down Syndrome	3 (2.7)
• Others	17 (15)
**Pre-existing central line (%)**	
• Percutaneous	14 (12.4)
• Surgical	21 (18.6)
**Increase of pSOFA score since admission (%)**	
• ≤ 4	62 (54.9)
• > 4	51 (45.1)
**Primary site of infection (%)**	
Bloodstream infection	
• - Bacteremia	30 (26.5)
• -Fungemia	2 (1.8)
• Respiratory tract	65 (57.5)
• Central nervous system	6 (5.3)
• Urinary tract	3 (2.7)
• Skin and soft tissue	3 (2.7)
• Others^a^	4 (3.5)

^a^Other sites of infection include 3 patients with Gastrointestinal symptoms and 1 patient with arthritis

Cultures and antigen testings were positive in 67.3% of patients (*n* = 76), and sepsis was caused by a bacterial pathogen in 60.5% (46/76). However, there was no significant difference in mortality between patients with and without bacterial infections (11.8% (9/76) vs 15.8% (12/76), respectively; *p* = 0.651). The rate of multi-drug resistant (MDR) bacteria was 21.7% (10/46). No significant difference was found concerning mortality between patients who were infected with MDR organisms (2.2% [1/46]) versus non-MDR organisms (23.9% [11/46]) (*p* = 0.252). Viruses and fungi accounted for 21.2 and 4.4% of all infections, respectively. The identified organisms among septic patients are shown in Additional file 2.

More than half of the patients (58.4%) received antibiotics within 3 h of admission, and 47.7% received fluid resuscitation up to 60 ml/kg. Respiratory support in the form of invasive mechanical ventilation was required in 58.5% of patients with a median duration of 8 days. Approximately half of the patients (50.44%) were diagnosed with septic shock. The 28-day mortality rate was 23.89% and the 7-day mortality rate was 10%. The median duration of PICU stay was 8 days. Table 2 describes the interventions performed for septic patients.

**Table 2:** Descriptive Statistics of interventions done to patients admitted to the Pediatric Intensive Care Unit (PICU) with sepsis

Variables	***n*** = 113
**Time to Start antibiotics in hours (%)**	
• < 3 h	66 (58.4)
• > 3 h (late admistration)	6 (5.3)
• Not documented	41 (36.3)
**Resuscitation Fluid Volume (%)**	
• 0 ml/kg	47 (41.6)
• Up to 60 ml/kg	54 (47.8)
• > 60 ml/kg	12 (10.6)
**Median Duration on Ionotropic Support in days (IQR)**	1 (4)
**Respiratory support (%)**	
• Nasal Cannula or Room-air	30 (26.5)
• Non-invasive	17 (15)
• Invasive	66 (58.5)

Univariate logistic regression was conducted to identify variables associated with overall mortality **(**Table 3**)**. Only an increase in pSOFA score >  4, pre-existing PIC line, mechanical ventilation, and PICU length of stay were significantly associated with increased likelihood of mortality (*p* < 0.05). These variables were entered into a multivariate regression analysis to obtain adjusted odds. Consequently, pre-existing PIC line and an increase in pSOFA score >  4 were significantly associated with increased mortality, with calculated ORs of 9.27 (95% CI: 1.28–67.29; *p* = 0.028) and 3.6 (95% CI: 1.30–9.93; *p* = 0.013), respectively (Table 4**).**

**Table 3:** Univariate analysis of risk factors associated with increased mortality among patients admitted to the Pediatric Intensive Care Unit (PICU) with sepsis

Variables	Outcome		*p*-value
	Alive *n* = 86	Dead *n* = 27	
**Age group**			0.502
1 month to < 1 year	28 (32.6%)	8 (29.6%)	
1 year to < 6 years	28 (32.6%)	12 (29.6%)	
6 to 14 years	30 (34.9%)	7 (25.9%)	
**Female Gender**	41 (47.7%)	13 (48.1%)	0.996
**Increase of pSOFA Score > 4 since admission**	33 (38.4%)	18 (66.67%)	0.012
**Pre-existing percutaneous central line (compared to surgical line only)**	6 (7%)	8 (29.6%)	0.006
**Any morbidity at admission**	61 (70.9%)	24 (89.9)	0.059
**Malignancy alone as a co-morbidity**	24 (27.9%)	9 (33.3%)	0.589
**Septic Shock**	39 (45.3%)	18 (66.7%)	0.053
**Giving any amount of Resuscitation Fluids**	48 (55.8%)	18 (66.7%)	0.318
**Ventilation (non-invasive vs. invasive only)**	41 (62.1%)	25 (92.65%)	0.020
**Late administration of Antibiotics**	4 (7.5%)	2 (7.4%)	0.687
**Median duration of PICU in days**^a^ **(IQR)**	8 (10)	11 (22)	0.031

^a^Cox & Snell R^2^ = 0.042

**Table 4:** Multivariate analysis of risk factors associated with increased mortality among patients admitted to the Pediatric Intensive Care Unit (PICU) with sepsis

Variables	Outcome		Crude Odds Ratio (CI)	Adjusted Odds Ratio (CI)	*p*-value
	Alive *n* = 86	Dead *n* = 27			
**Increase of pSOFA Score > 4 since admission**	33 (38.4%)	18 (66.67%)	3.21 (1.29–7.98)	3.6 (1.30–9.93)	0.013
**Pre-existing percutaneous central line (compared to surgical line only)**	6 (7%)	8 (29.6%)	12.67 (2.09–76.70)	9.27 (1.28–67.29)	0.028
**Non-invasive ventilation (compared to invasive only)**	41 (62.1%)	25 (92.65%)	6.034 (1.33–27.0)	4.30 (0.82–22.62)	0.085
**Median duration of PICU stay in days (IQR)**	8 (10)	11 (22)	1.04 (1.003–1.07)	1.02 (0.982–1.06)	0.347

## Discussion

This study demonstrated a high pediatric sepsis mortality rate, comparable to that of other studies performed in this region [8, 9]. Risk factors associated with increased mortality were an increase in *pSOFA score* > 4 points and the presence of a pre-existing PIC line. Most patients included in the study had associated comorbidities, including malignancies with no association to increased risk of mortality. In addition, delayed administration of antibiotics was not found to increase the risk of mortality.

The reported pediatric sepsis mortality rate varies significantly among different studies, particularly between developed and developing countries. A study conducted by Hassan et al. to assess the feasibility and efficacy of sepsis management guidelines in the PICU revealed a mortality rate of 47.8 and 26.2%, before and after implementing the guidelines, respectively [8]. Another study conducted in a tertiary PICU in Riyadh reported an overall mortality rate of 17.9% for severe sepsis and septic shock [6]. Moreover, in Egypt, the mortality rate of sepsis has been reported as 28.1 and 8.8% by Bekhit et al. and El-Mashad et al., respectively [7, 9]. A study performed by the Italian Pediatric Sepsis Study group in Italian PICUs reported a mortality rate of 17.8% for severe sepsis [12]. Xiao C et al. carried out an epidemiological study in the main PICU centers in southwest China, reporting a severe sepsis mortality rate of 18.8% [13]. In 2014, Wang et al. reported a higher rate (34.6%) for severe sepsis and septic shock among Chinese children [14]. This study’s high mortality rate may be related to the use of the pSOFA score instead of the SIRS criteria and thus enrolling higher number of sick patients. Similar to other studies, an increase in the pSOFA score > 4 points was independently associated with increased risk of mortality. This indicates that a higher pSOFA score suggests increased organ dysfunction and therefore pSOFA score can be a useful predictor of mortality [11, 15].

Percutaneous central venous catheters are common sites of bacterial entry and are, thus, associated with an increased risk of central line-associated bloodstream infections [16]. Patients with these lines are at high risk of bloodstream infections due to aggressive hospital-acquired organisms. This type of catheter is usually only required for very sick patients with significant comorbidities, further explaining their association with increased risk of mortality. There was no difference in mortality rate between patients with early versus delayed antibiotic administration, which could be attributed to the small number of enrolled patients and the underpowered design of the study. However, a study conducted by Alsadoon et al. in Riyadh, Saudi Arabia reported delayed antibiotic administration was not significantly associated with higher PICU mortality in children diagnosed with severe sepsis [6].

The respiratory tract was the most common primary location of infection, followed by the bloodstream and the central nervous system, as seen in previous studies [5, 13, 17]. The etiologic pathogens of this study were consistent with those of other studies; bacterial infections were the most common, followed by viral and fungal infections [5, 12, 13]. Nonetheless, this study was unable to reveal a relationship between isolated bacteria or MDR organisms and increased risk of mortality.

This study used the age-adapted Sepsis-3 criteria, which uses pSOFA scores rather than the SIRS criteria to identify patients diagnosed with sepsis. The SIRS criteria lack specificity for identifying children with infection at a substantially higher risk of mortality [18]. Studies performed by Schlapbach et al. in Australia and New Zealand and El-Mashad et al. in Egypt – Middle East and North Africa region demonstrated that adapting Sepsis-3 to age-specific criteria performs better than the SIRS criteria in differentiating children with infection at a substantially higher mortality risk. Our findings support the translation of Sepsis-3 into pediatric-specific sepsis definitions and highlight the importance of robust pediatric organ dysfunction characterization [10, 18]. Validation studies regarding age-adapted Sepsis-3 are promising, indicating that Sepsis-3 has the potential to be the future definition for pediatric sepsis [11].

KAMC-J is one of the major tertiary centers in the western region of Saudi Arabia. Thus, most of the children admitted to the pediatric ward have many comorbidities. The pediatric oncology section is the most used in the region, receiving a significant number of referrals; therefore, a high percentage of the children admitted to the PICU have malignancy as a co-morbidity. This explains the high number of patients with comorbidities enrolled in the study. Many studies have suggested patients with malignancies are more likely to die of sepsis than those without malignancies [12, 17]. Nevertheless, this study found no such association, which is comparable to other studies [19, 20]. This could be attributed to the early recognition and timely management of such vulnerable patients.

## Conclusions

The incidence of sepsis in KAMC-J is comparable to international data; however, the mortality rate remains high compared to developed countries. Following international guidelines for central line insertion and maintenance is of paramount importance. Implementation of a national database will help conduct large-scale studies to determine areas of weakness regarding the diagnosis of sepsis and, therefore, improve pediatric sepsis outcomes.

### Limitations

The study was limited by its retrospective design and the relatively small number of patients from a single center. Some of the data concerning the timing of administration of the antibiotics was missing in some of the charts, which probably affected the univariate analysis of the variable. The limited number in the sub-groups of the multivariate regression underpowered the adjusted odds ration of the variables included in the model.

## Supplementary Information

**Additional file 1.**

**Additional file 2.**

## Data Availability

The datasets used and/or analyzed during the current study are available from the corresponding author on reasonable request.

## Abbreviations

PICU: Pediatric intensive care unit
KAMC-J: King Abdulaziz Medical City- Jeddah
pSOFA: Pediatric sequential organ failure assessment score
PIC line: Peripherally inserted central line
OR: Odds ratio
CI: Confidence interval
MDR: Multi-drug resistance
