# Supplementary material for: Epidemiology of pediatric sepsis in the pediatric intensive care unit of king Abdulaziz Medical City, Jeddah, Saudi Arabia
Source: BMC Pediatr. 2021 May 7;21:222. doi: 10.1186/s12887-021-02686-0 (PMC8103596; doi:10.1186/s12887-021-02686-0)
Supplement: Supplementary file 1 — Additional file 1. [file 12887_2021_2686_MOESM1_ESM.docx]

| **Appendix 1:** The adapted Pediatric Sequential Organ Failure Assessment Score (pSOFA)^⁋^ | | | | | |
| --- | --- | --- | --- | --- | --- |
| **Variables** | Score^a^ | | | | |
|  | **0** | **1** | **2** | **3** | **4** |
| **Respiratory** | | | | | |
| PaO_2_:FiO_2_^b^  or  SpO_2_:FiO_2_^c^ | ≥400 | 300-399 | 200-299 | 100-199 With  respiratory support | <100 With  respiratory support |
|  | ≥292 | 264-291 | 221-264 | 148-220 With respiratory support | <148 With  respiratory support |
| **Coagulation** | | | | | |
| Platelet count,  ×103/μL | ≥150 | 100-149 | 50-99 | 20-49 | <20 |
| **Hepatic** | | | | | |
| Bilirubin, mg/dL | <1.2 | 1.2-1.9 | 2.0-5.9 | 6.0-11.9 | >12.0 |
| **Cardiovascular** | | | | | |
| MAP by age group or vasoactive infusion, mm Hg or μg/kg/min^d^ | | | | | |
| <1 month | ≥46 | <46 | Dopamine  hydrochloride ≤5  or dobutamine  hydrochloride  (any) | Dopamine  hydrochloride >5 or  epinephrine ≤0.1 or  norepinephrine  bitartrate ≤0.1 | Dopamine hydrochloride >15 or  epinephrine >0.1 or  norepinephrine  bitartrate >0.1 |
| 1-11 months | ≥55 | <55 |  |  |  |
| 12-23 months | ≥60 | <60 |  |  |  |
| 24-59 months | ≥62 | <62 |  |  |  |
| 60-143 months | ≥65 | <65 |  |  |  |
| 144-216 months | ≥67 | <67 |  |  |  |
| >216 months^e^ | ≥70 | <70 |  |  |  |
| **Neurologic** | | | | | |
| Glasgow Coma  Score^f^ | 15 | 13-14 | 10-12 | 6-9 | <6 |
| **Renal** | | | | | |
| Creatinine by age group, mg/dL | | | | | |
| <1 month | <0.8 | 0.8-0.9 | 1.0-1.1 | 1.2-1.5 | ≥1.6 |
| 1-11 months | <0.3 | 0.3-0.4 | 0.5-0.7 | 0.8-1.1 | ≥1.2 |
| 12-23 months | <0.4 | 0.4-0.5 | 0.6-1.0 | 1.1-1.4 | ≥1.5 |
| 24-59 months | <0.6 | 0.6-0.8 | 0.9-1.5 | 1.6-2.2 | ≥2.3 |
| 60-143 months | <0.7 | 0.7-1.0 | 1.1-1.7 | 1.8-2.5 | ≥2.6 |
| 144-216 months | <1.0 | 1.0-1.6 | 1.7-2.8 | 2.9-4.1 | ≥4.2 |
| >216 months^e^ | <1.2 | 1.2-1.9 | 2.0-3.4 | 3.5-4.9 | ≥5 |
| **Abbreviations:** FiO_2_, fraction of inspired oxygen; MAP, mean arterial pressure; pSOFA, pediatric Sequential Organ Failure Assessment; SpO_2_, peripheral oxygen saturation.  SI conversion factors: To convert bilirubin to micromoles per liter, multiply by 17.104; creatinine to micromoles per liter, multiply by 88.4; and platelet count to ×109/L, multiply by 1.  ^⁋^ Available from: Matics TJ, Sanchez-Pinto LN. Adaptation and validation of a pediatric sequential organ failure assessment score and evaluation of the sepsis-3 definitions in critically ill children. JAMA pediatrics. 2017 Oct 1;171(10):e172352-.  ^a^ The pSOFA score was calculated for every 24-hour period. The worst value for every variable in each 24-hour period was used to calculate the subscore for each of the 6 organ systems. If a variable was not recorded in a given 24-hour period, it was assumed to be normal and a score of 0 was used. Daily pSOFA score was the sum of the 6 subscores (range, 0-24 points; higher scores indicate a worse outcome).  ^b^ PaO2 was measured in millimeters of mercury.  ^c^ Only SpO2 measurements of 97% or lower were used in the calculation.  ^d^ MAP (measured in millimeters of mercury) was used for scores 0 and 1; vasoactive infusion (measured in micrograms per kilogram per minute), for scores 2 to 4. Maximum continuous vasoactive infusion was administered for at least 1 hour.  ^e^ Cutoffs for patients older than 18 years (216 months) were identical to the original SOFA score.  ^f^ Glasgow Coma Scale was calculated using the pediatric scale. | | | | | |
