# Supplementary material for: Epidemiology of pediatric sepsis in the pediatric intensive care unit of king Abdulaziz Medical City, Jeddah, Saudi Arabia
Source: BMC Pediatr. 2021 May 7;21:222. doi: 10.1186/s12887-021-02686-0 (PMC8103596; doi:10.1186/s12887-021-02686-0)
Supplement: Supplementary file 2 — Additional file 2. [file 12887_2021_2686_MOESM2_ESM.docx]

| **Appendix 2:** Culture results among septic patients from January 1, 2013, to December 31, 2017 | |
| --- | --- |
| **Variables** | **n=113 (%)** |
| No Organism Identified | **37 (32.7)** |
| **Gram-negative organisms***  *Pseudomonas aeruginosa*  *Escherichia coli*  *Acinetobacter baumanni*  *Enterobacter cloacae,*  *Klebsiella* *pneumoniae*  *Salmonella typhi*  *Serratia marcescens*  *Stenotrophomonas maltophilia* | **21 (18.6)**  7  6  3  1  1  1  1  1 |
| **Gram-positive organisms**  *Staphylococcus species*  *- Methicillin resistant*  *- Methicillin sensitive*  *Streptococcus pneumonia*  *Coagulase negative staphylococci*  *Bordetella pertussis*  *Moraxella catarrhalis* | **12 (10.6)**  3  2  3  2  1  1 |
| **Viruses**  Respiratory syncytial virus  Influenza  Varicella zoster | **25 (21.2)**  18  6  1 |
| **Fungi**  *Trichosporon Asahii*  *Candida Albicans*  *Aspergillus* | **5 (4.4)**  2  2  1 |
| **Multiple organisms identified**** | **13 (11.5)** |
| **Multi-drug resistant* ***(MDR****) organisms were 1 Escherichia coli, 1 Stenotrophomonas maltophilia, 3 Acinetobacter baumanni, 1 Enterobacter cloacae, and 1 Klebsiella* *pneumoniae.*  ***It included 1 MDR Escherichia coli, 1 MDR Pseudomonas aeruginosa, and 1 MDR Serratia marcescens.* | |
